# Tailoring the properties of quantum dot-micropillars by ultrafast optical injection of free charge carriers

**DOI:** 10.1038/s41377-021-00654-y

**Published:** 2021-10-19

**Authors:** Emanuel Peinke, Tobias Sattler, Guilherme M. Torelly, Patricia L. Souza, Sylvain Perret, Joël Bleuse, Julien Claudon, Willem L. Vos, Jean-Michel Gérard

**Affiliations:** 1 Université Grenoble Alpes, CEA, IRIG-PHELIQS, “Nanophysique et Semiconducteurs” group, F-38000 Grenoble, France; 2 LabSem-CETUC, Pontifícia Universidade Católica do Rio de Janeiro, Rio de Janeiro, 22451-900 Brazil; 3 Complex Photonic Systems (COPS), MESA+ Institute for Nanotechnology, University of Twente, P.O. Box 217, 7500 AE Enschede, the Netherlands

**Keywords:** Quantum dots, Nanocavities

## Abstract

We review recent studies of cavity switching induced by the optical injection of free carriers in micropillar cavities containing quantum dots. Using the quantum dots as a broadband internal light source and a streak camera as detector, we track the resonance frequencies for a large set of modes with picosecond time resolution. We report a record-fast switch-on time constant (1.5 ps) and observe major transient modifications of the modal structure of the micropillar on the 10 ps time scale: mode crossings are induced by a focused symmetric injection of free carriers, while a lifting of several mode degeneracies is observed when off-axis injection breaks the rotational symmetry of the micropillar. We show theoretically and experimentally that cavity switching can be used to tailor the dynamic properties of the coupled QD–cavity system. We report the generation of ultrashort spontaneous emission pulses (as short as 6 ps duration) by a collection of frequency-selected QDs in a switched pillar microcavity. These pulses display a very small coherence length, attractive for ultrafast speckle-free imaging. Moreover, the control of QD-mode coupling on the 10 ps time scale establishes cavity switching as an appealing resource for quantum photonics.

## Introduction

In the context of the blossoming development of nanophotonics, photonic micro and nanostructures have become powerful tools to tailor the optical properties of semiconductor emitters in solid-state cavity quantum electrodynamics (CQED) experiments^1–5^. The strong coupling regime has been observed for quantum wells in planar cavities^6^, and for a single quantum dot (QD) coupled to a single-cavity mode^5^. In the weak coupling regime, the enhancement^1,3,7^ and the inhibition^2,8–10^ of the spontaneous emission (SpE) rate have been observed for QDs. These drastic modifications of the emitter’s behavior result from the engineering of the modes of the electromagnetic field and of the emitter-mode coupling. These key fundamental advances have fueled the development of a novel class of optoelectronic devices exploiting CQED effects on a single QD, including single-mode sources of single photons^1,3,4,11–15^ or sources of entangled photon pairs^16,17^.

Although solid-state CQED has initially been inspired by pioneering atomic-CQED, there is still a major difference between these two classes of experiments. In atomic-CQED, one can control the interaction time between a flying atom and a cavity mode by varying the atom velocity^18^. By contrast, the vast majority of solid-state CQED experiments to date are “static” experiments, during which the emitter-mode coupling is kept constant for the entire duration of SpE events^1–17^.

In the quest for “dynamic” solid-state CQED, several groups have recently proposed strategies to rapidly modify the emitter-field coupling, “rapidly” meaning within a time that is shorter than the relevant time scale for the static coupled system (emitter lifetime in the weak coupling regime, Rabi oscillation period in the strong coupling regime). One can for instance induce ultrafast modifications of the local density of optical states (LDOS) at the emitter location using short laser pulses to change the refractive index of the constituent material. It is possible in principle to change the spectral detuning of an emitter with respect to the band-edge of a photonic crystal^19–24^ or to a discrete cavity mode^25^. In a long-term perspective, a dynamic control of CQED effects is obviously attractive, e.g., to switch-on and -off Rabi oscillations, and prepare Fock states in cavity modes^26^, in a way that would resemble the operation of micromasers based on flying Rydberg atoms^18^. It could also be used to tune the magnitude of the Purcell effect and tailor the temporal envelope of single-photon pulses. This capability is highly desirable in view of applications to photonic quantum computing and simulations^27^ and quantum state transfer through photon exchange^28^.

Various approaches towards a fast control of emitter-cavity detuning have been explored. The electrostatically induced Stark effect has been considered for trapped ions^29^, as well as QDs^30,31^, for adjusting the emitter frequency with respect to a target cavity mode. In the latter case, its dynamical range happens to be limited to the sub-GHz range^31^, which is not sufficient for controlling CQED effects on QDs. One can alternatively tune the frequency of a mode with respect to a target emitter. Transient changes of the frequency of cavity modes on the few-10 ps time scale have been induced in planar and photonic crystal cavities using pulses of acoustic waves^32,33^.

All-optical cavity switching provides an even faster and more versatile means. The reversible frequency switching of cavity modes can be achieved on a sub-picosecond time scale using the electronic Kerr effect^34–36^. The mainstream approach, though, relies on the optical injection of free carriers using a pulsed laser pump to change the refractive index of the semiconductor layer forming the microcavity^37–52^. While an ultrafast “switch-on” behavior is observed on the few-ps time scale, the relaxation of the mode towards its original frequency is usually slower, as it is governed by free carrier recombination. Nevertheless, “switch-off” time constants in the sub-100 ps range can be obtained in small cavities thanks to fast non-radiative recombination at etched surfaces, fast diffusion out of the cavity mode^50^, or by engineering the bulk non-radiative recombination rate (e.g., by growing parts of the semiconductor microcavity at a lower-than-standard substrate temperature^49,51^). This latter switching scheme is very well suited for the dynamic control of QD–cavity systems in the weak coupling regime, since the free carrier lifetime ranges between a few tens of ps and a few ns. A reversible change of the SpE rate of a QD in a photonic crystal cavity on the 200 ps time scale was recently demonstrated using this approach^47^. The ultrafast injection of free carriers is exploited in various contexts to change cavity mode properties, such as their frequency, in view of color-change experiments^39–43^, the cavity Q, to catch and release light pulses^44^, the coupling and photon exchange between distant cavities^45^, or the modes field distributions in coupled cavity systems^46,47^.

We review in this paper recent advances towards dynamic solid-state CQED, which have been obtained by the PHELIQS laboratory in Grenoble, using GaAs/AlAs pillar microcavities containing InAs QDs—dubbed as quantum dot-micropillars (QDMs)—as main workhorse, and optical injection of free charge carriers as cavity switching method. For all results presented in this review, we use micropillars containing a one-wavelength-thick GaAs cavity layer, surrounded by two distributed Bragg reflectors (15 periods on the top side, 25 periods on the bottom side). A detailed presentation of the QDM samples used for experiments is given in the Supplementary Information (SI).

We first show in section “Probing switching events with QDs as an internal light source” that QD ensembles can be used as a broadband internal source to light up all cavity modes during switching events^48^. Using a detection set-up combining a spectrometer and a streak camera, we track the frequencies of many cavity modes in parallel, with a 2 ps time resolution. This fast and convenient characterization method enables extensive studies of the switching behavior as a function of the parameters of the laser pump (wavelength, pulse energy, beam shape). For conditions that ensure a spatially uniform refractive index change, novel insights are obtained on the dynamics of the switching behavior of the fundamental mode after a pump pulse. Under non-uniform carrier injection, using a focused pump beam, mode-dependent switching behaviors are observed. Amazing transient features are witnessed, noticeably a reordering of the modes of the micropillar under symmetric carrier injection respective to the pillar axis, and a lifting of the polarization degeneracy of some modes when one uses an off-axis carrier injection to break the circular symmetry of the pillar microcavity. A model taking into account free carrier diffusion and recombination gives a good quantitative modeling of the markedly different switching behaviors for a large set of cavity modes.

Section “Controlling SpE dynamics using cavity switching: insights from theory” is devoted to the presentation and theoretical modeling of two possible applications of cavity switching in the field of quantum photonics. By inducing a transient coupling between an emitter and a cavity mode, one can switch on and off the Purcell effect in a fast way. The system then generates a SpE burst whose duration is much shorter than the lifetime of the uncoupled emitter. On the basis of a rate equation analysis, we introduce and compare possible signatures of “Purcell switching” in such experiments. However, besides on/off switching, one could also play more smoothly on the detuning between the emitter and a cavity mode. Solving quantum Langevin equation, we show that single-photon pulses with tailored temporal envelopes can be produced by state-of-the-art QD–cavity systems with a high efficiency and a high fidelity with respect to the target pulse^53^.

In section “Preparation of ultrashort low-coherence pulses of SpE using switched QDM”, we use cavity switching to tune on and off, and on the few-ps time scale, the coupling between QDs and the fundamental mode of a pillar microcavity. In these experiments, a set of spectrally selected QDs enters into resonance with the fundamental mode, as it relaxes towards its original frequency after a switch. This approach is used to generate pulses as short as 6 ps^54,55^. Unlike laser pulses, these short SpE pulses display a very weak spectral coherence.

Finally, we briefly discuss some perspectives opened by this work in section “Some perspectives related to this work”.

## Probing switching events with QDs as an internal light source

Practical applications of cavity switching in the field of quantum photonics require a detailed knowledge of the amplitude and dynamics of the frequency shifts of the modes. Since the first switching studies, performed on micropillars in the '80s in the context of the development of bistable optical devices^37,38^, ultrafast pump–probe spectroscopy in reflection geometry has been the reference technique for probing switching events^34–36,49–52^. This technique provides excellent time resolution^56^, but is relatively cumbersome (two synchronized tunable lasers are needed), and relatively slow due to the pointwise exploration of the 2D frequency-time space, which hinders extensive parametric studies.

Furthermore, one is able to probe by transmission or reflection spectroscopy only modes that have a non-zero overlap with the probe beam. As an example, for a Gaussian beam whose waist is matched to the top facet of a micropillar, one only detects the fundamental cavity mode in the reflection or transmission curves^57^. Using a tightly focused beam with a waist smaller than the cavity, alleviates this constraint to some extent, and permits probing additional modes^58,59^.

It is nowadays well known that one can systematically study all modes of a static cavity in a simple photoluminescence (PL) experiment, by using a collection of embedded QDs as a broadband internal light source^1,60^. In the SpE regime, QDs behave as independent point-like emitters and light up systematically all cavity modes within the broad frequency range covered by their inhomogeneously broadened emission line. Several attempts have been made to adapt this approach to the characterization of switched single-mode photonic crystal cavities^61^ and micropillars^55^. In these experiments, the same laser pulse was used both to excite embedded InAs QDs and to switch the cavity modes through free carrier injection in GaAs layers. There is in turn some delay (~25 ps) in the onset of the QD PL due to carrier capture and relaxation processes. The PL is too weak for studying the switch-on behavior of the cavity modes during the first ten to fifteen picoseconds after the pump pulse.

We have recently improved this approach by introducing a two-pulse pumping sequence to ensure that the internal light source is bright enough to probe the entire switching event. As detailed in Sattler et al. ^48^, we use a fibered system to split the pulses delivered at 76 MHz by a Ti:sapphire laser into a weak pulse, which lights up the QDs but does not induce significant switching, and a stronger one, which is delayed with respect to the first one and switches the cavity. We use a detection set-up combining a grating spectrometer and a streak camera to track in parallel the time-dependent frequencies of many resonant modes, over the entire switching event with a ps time resolution. QD growth conditions are chosen so that the QD ensemble emission is centered at around 1.36 eV, with a 45 meV full-width at half-maximum (FWHM). This emission range is well suited for an efficient detection by the photocathode of our streak camera (see SI).

**Fig. 1:**
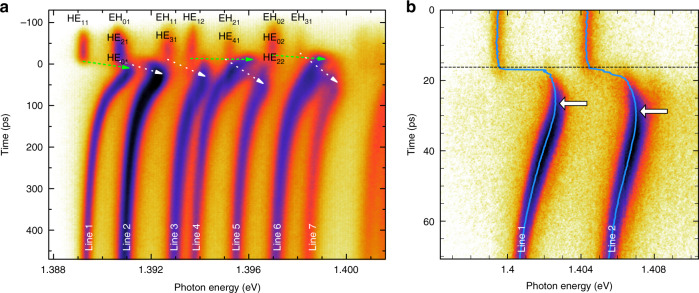
Typical streak camera images obtained for two different QDMs and different operating time ranges for an on-axis pump beam. **a** A 500 ps-long time range enables capturing the entire switching event for the seven first lines of a 5 µm diameter QDM (1.5 µm diameter pump centered on the pillar axis, pump photon energy *E*_*p*_ = 1.57 eV, around 10 pJ/pulse). The arrows are guides to the eye, showing the shift of the lines during the switch-on event. The arrow’s tips point at the time-frequency point corresponding to the maximum line shift. The names of all pillar modes contributing to an emission line are indicated at the position of lines prior to the switch. **b** The two first lines of a 3 µm diameter QDM are studied with a 2 ps resolution over a 70 ps-long time range (pump waist matched to the top facet of the QDM, *E*_*p*_ = 1.55 eV, 7 pJ/pulse). The blue line represents the time-dependent position of the modes, deduced from Lorentzian fits of the PL spectrum at all times. The white arrows point at the maximum blue shift for each line.

We display in Fig. 1 two streak camera images, which illustrate the complementarity of experiments performed over different time ranges (TR). Using a 600 ps-long TR, one can capture the entire event including full relaxation of the modes. Before the pump pulse at around *t* = 0, the frequencies of the emission lines do not change, which confirms that possible small switching effects induced by the previous pulse have faded away. These frequencies correspond therefore to the ones of the modes of the unswitched QDM, as observed in cw PL experiments, and serve as references to evaluate switching effects. Emission lines are labeled using the standard names of all pillar modes, which contribute to each line^60^. At around *t* = 0, the injection of free carriers induces locally a negative change of the refractive index of GaAs layers. As a consequence, all lines experience a blue shift, within less than 30 ps, albeit with significantly different switching amplitudes and time delays before reaching the maximum shift. Operating in this TR, the time resolution is not high enough to track precisely the modes during the switch-on. After around 100 ps, all lines relax back towards their reference frequency and exhibit a common behavior, which can be described by an exponential law, with a 200 ps characteristic time constant that corresponds to the decay time of the population of free electron–hole pairs.

By contrast, our set-up has a time resolution better than 2 ps when using the streak camera in its shortest TR mode, which enables probing the ultrafast transient effects that occur during and just after the switch, as shown by Fig. 1b and other examples to be presented below.

One can easily explain switching effects in a qualitative way by considering the evolution of the population of free carriers and their impact on the transverse refractive index profile in the micropillar. Since the pump photon energy *E*_*p*_ is above the GaAs bandgap (~1.52 eV at 4 K), charge carriers are photogenerated with excess energy in the conduction and valence bands of GaAs. They then relax within a few ps^62^ and provide a maximal change of the refractive index once relaxation toward the band extrema of GaAs is completed^63^. Further evolution of the distribution of free carriers is governed by lateral diffusion on the few-10 ps scale and carrier recombination. While population decay at long times is always dominated by non-radiative recombination at etched sidewall surfaces, radiative recombination of electrons and holes can contribute to it at shorter delays, when the free carrier density is the largest.

Being both fast and precise, this characterization method makes parametric studies much easier than previous techniques. It allows, for instance, the detailed study of the switch-on dynamics by analyzing images such as that shown in Fig. 1b. By optimizing the pump power, we could observe a rise-time constant around 1.5 ps^48^, significantly smaller than previously reported values^49–52^. This is obviously interesting in view of the ultrafast control of the spectral detuning between an artificial atom and one- or few-cavity modes.

**Fig. 2:**
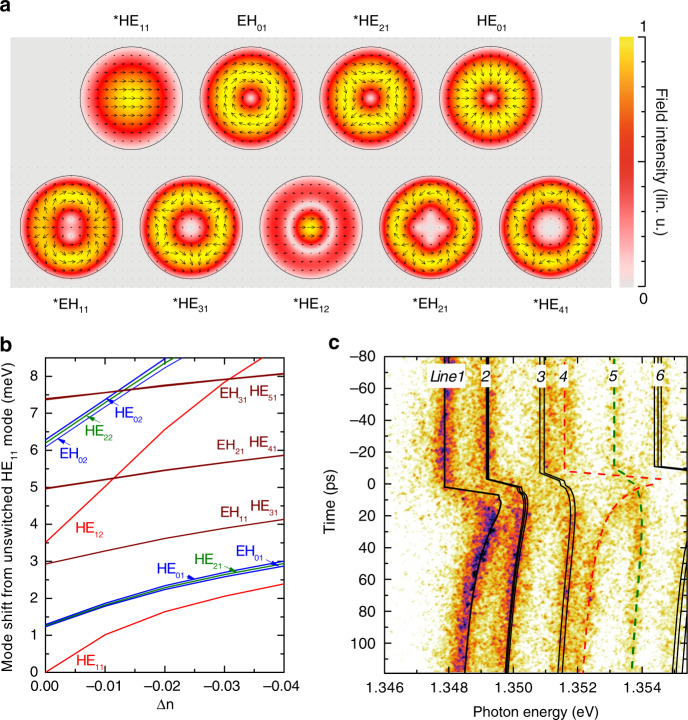
Mode crossings induced by on-axis free carrier injection. **a** Vectorial maps of the in-plane electric field and color maps of the field intensity for the confined modes which contribute to Lines 1–5 in a 6 µm diameter unswitched QDM (Line 1: HE_11_; Line 2: HE_01_, HE_21_, EH_01_; Line 3: HE_31_, EH_11_; Line 4: HE_12_; Line 5: HE_41_ and EH_21_). A black line marks the edge of the QDM and the linear color scale is normalized at the antinode for each mode. For two-fold polarization-degenerate modes, labeled with a star, only one map is shown. **b** Frequencies of the modes of a 6 µm diameter QDM, after injection of a Gaussian centered distribution of free carriers with 2 µm FWHM, as a function of Δ*n*, the maximum refractive index change induced in GaAs layers (i.e., on the pillar axis). The frequency of mode HE_11_ in the unswitched QDM is used as reference. **c** Streak camera image obtained on a 6 µm diameter switched QDM excited by a focused centered pump beam. Here, *E*_*p*_ = 1.65 eV and *P* = 20 pJ/pulse. The Gaussian pump beam has a 2 μm diameter waist. The lines show the calculated mode frequencies, resulting from a model taking into account carrier injection, lateral diffusion, and recombination^48^, assuming a refractive index change Δ*n* = −0.02 at beam center in the GaAs cavity layer just after excitation by the pump pulse.

One can also easily explore the huge impact of the spatial characteristics of the pump beam (i.e., beam diameter and position with respect to the pillar axis) on switching features. In micropillars, the resonant modes have amazingly different transverse field distributions as shown in Fig. 2. For instance, the fundamental mode HE_11_, which gives rise to Line 1 in Fig. 1b has no node and its antinode is on the pillar axis. By contrast, all modes contributing to Line 2, also shown in Fig. 1, have a donut shape and zero intensity on the pillar axis. These differences, which are inherited from the properties of guided modes in cylindrical waveguides^64^, and are well known from theory, have also been clearly confirmed by mode intensity mapping, using reflection spectroscopy with a tightly focused white beam^59^.

Consequently, one observes drastically different switching behaviors depending on the size and location of the pump beam with respect to the pillar top facet. If one uses a pump beam much broader than the pillar diameter, uniform carrier injection is realized and all modes display the same switching amplitude and the same switching dynamics^48^. On the opposite, one can observe mode-specific switching behaviors for a non-uniform carrier injection. As first shown by pump-probe reflection spectroscopy experiments^52^, and as confirmed by the time-resolved PL results shown in Fig. 1b, Lines 1 and 2 behave differently when the pump spot matches the pillar top facet. Thanks to a better overlap between the Gaussian initial distribution of free carriers and the mode profile, Line 1 exhibits a slightly larger switching amplitude and a faster switch-on dynamics. For Line 2, the largest switching amplitude is only obtained after lateral free carrier redistribution, which induces some delay (see red arrows in Fig. 1b).

One can expect more spectacular “differential switching” features when the pump spot is much smaller than the pillar top facet. For a focused and centered pump beam, we expect to observe (according to mode perturbation theory, see, e.g., Vassalo^65^) a highly sensitive and selective response of the modes whose main antinode is located on the pillar axis. Within the set of modes shown in Fig. 2a (giving rise to Lines 1–5) this is only the case for the modes HE_1*n*_; additionally, the field is more concentrated around the pillar axis for HE_12_ than for HE_11_, which should warrant to HE_12_ the highest sensitivity to optically induced refractive index changes. This is confirmed by the numerical simulation of the expected mode blue shifts after excitation by a focused Gaussian beam, which are plotted in Fig. 2b as a function of the maximum index change, induced in GaAs at the center of the pump spot. HE_12_ is the most sensitive to such a perturbation and shifts twice as much as the fundamental mode HE_11_. We assume for these calculations a linear dependence of the refractive index change as a function of the carrier density, which is reasonable approximation for carrier densities^62^ above 10^17^ cm^−3^ that are typically used in switching experiments.

Since the switch does not break circular symmetry, it does not induce any coupling between modes that have different symmetry degrees (i.e., for instance different azimuthal orders *m*, or different behaviors with respect to a mirror symmetry). As shown in Fig. 2b, one expects therefore to observe mode crossings (instead of anti-crossings) when the perturbation strength is increased. This is noticeably the case for the highly sensitive mode HE_12_ (related to Line 4), which crosses the weakly sensitive donut-shape modes EH_11_ and HE_31_ (which give rise to Line 5) for | Δ*n* | > 0.1.

This theoretical prediction is validated through the observation of a transient re-ordering of resonant pillar modes just after the switch in several experiments. In Fig. 1a, we see that Lines 4 and 6 (both related to modes with a large field intensity close to the pillar axis) experience a large switch within few ps. For Line 4, the shift is larger than the separation of Lines 4 and 5 before the switch. This is also the case for the shift of Line 6, when compared to the separation of Lines 6 and 7. By contrast, Lines 5 and 7, related to donut-shape modes, exhibit a relatively slow shift that reaches its maximum only after several tens of picoseconds, once free carriers have been fully redistributed laterally by diffusion. So, considering the spectral positions of Lines 4 and 6 before and after the switch, as well as the slow behavior of Lines 5 and 7, we can conclude that Lines 4 and 5, as well as Lines 6 and 7, have to cross twice at short delays.

We have recently attempted to study such a behavior over a narrower TR, so as to track precisely the modes during the reordering event. As shown by Fig. 2c, Lines 4 and 5 overlap about 10 ps after the switch, thanks to the large and fast switch of Line 4 (please see also Fig. S7 in the SI, showing the raw streak camera image). Due to free carrier diffusion away from the center of the pillar, Line 4 experiences a fast relaxation at longer delays, which restores the standard ordering and spectral separation of the modes. We do not observe any sign of anti-crossing behavior when Lines 4 and 5 overlap, in agreement with numerical simulations (see, e.g., Fig. 2b). We also show in Fig. 2c the calculated evolution of the mode frequencies during the switch, taking into account inhomogeneous carrier injection, carrier diffusion and recombination. This modeling, which gives a satisfying description of the temporal evolution of all modes at all times, suggests that Lines 4 and 5 actually cross and that a reverse ordering is obtained during about 10 ps. In this experiment, however, the broadening of the lines is similar to their frequency difference, which hinders a clear observation of the mode crossing and reordering. Experiments on higher-*Q* micropillars are under way to go beyond this promising but still preliminary result.

**Fig. 3:**
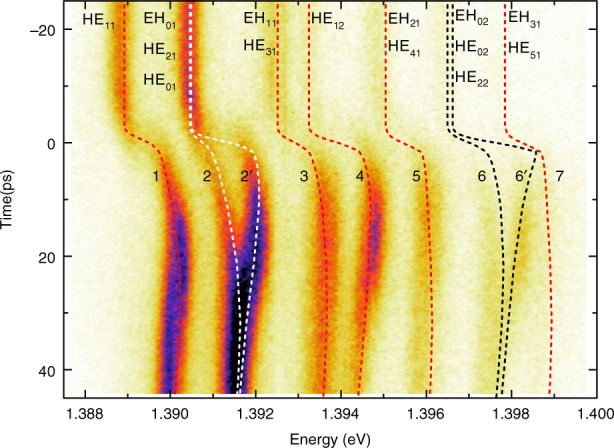
Mode splittings induced by a focused off-axis pump beam. The streak camera image is obtained for a 5.3 μm diameter QDM. Here, *E*_*p*_ = 1.55 eV and *P* = 10 pJ/pulse. The Gaussian pump beam has a 1.1 μm diameter at waist and is focused 0.9 μm away from the center of the QDM top facet. The dashed lines correspond to the calculated mode frequencies^48^ (for this simulation, the maximum refractive index change Δ*n* = −0.06). The splitting of Lines 2 and 6 due to the breaking of the circular symmetry of the QDM through asymmetric carrier injection is highlighted by this experiment.

Additionally, one can use an off-axis focused injection of free carriers to break the circular symmetry of the micropillar^48^. The result of such an experiment is shown in Fig. 3 for a 5 µm diameter QDM. Clear transient splittings are observed during around 20 ps after the switch for Lines 2 and 6, due to the shift from circular symmetry to planar symmetry. This amazing effect vanishes as soon as carrier diffusion restores the circular symmetry. The transient effects observed in Figs. 2c and 3 can be reproduced in detail for all modes by numerical simulations taking into account the carrier injection geometry, as well as carrier diffusion and recombination^48,52^. Importantly, such simulations rely on a single fitting parameter, Δ*n*, which is the maximum refractive index change of GaAs layers, induced just after the pulse at the center of the pump spot.

On the basis of this detailed understanding of switching events in in QDM, possible applications of cavity switching in the context of quantum photonics will be introduced in the following two sections.

## Controlling SpE dynamics using cavity switching: insights from theory

Solid-state optical microcavities such as micropillars and microdisks are not perfect “photonic dots”, as, besides the discrete resonant cavity modes, the continuum of non-resonant modes of the environment contributes to the LDOS inside the cavity. In the weak coupling regime, well-known benefits come from the coupling between an artificial atom such as a QD and a (static) cavity mode whose Purcell factor *F*_*p*_ is large enough (*F*_*p*_ > 1)^1–4,7^. The SpE rate into the cavity mode is selectively enhanced, while SpE into non-resonant modes is basically unchanged. On one side, SpE is accelerated; this is the well-known Purcell effect. On the other side, photons are preferentially funneled into the cavity modes. SpE becomes single mode for large values of *F*_*p*_. If the cavity mode has a directional emission pattern, as for e.g., the fundamental mode of pillar cavities, this improves strongly the collection of emitted photons. These three beneficial effects are noticeably exploited in QD-micropillar sources of single photons^11–15^.

**Fig. 4:**
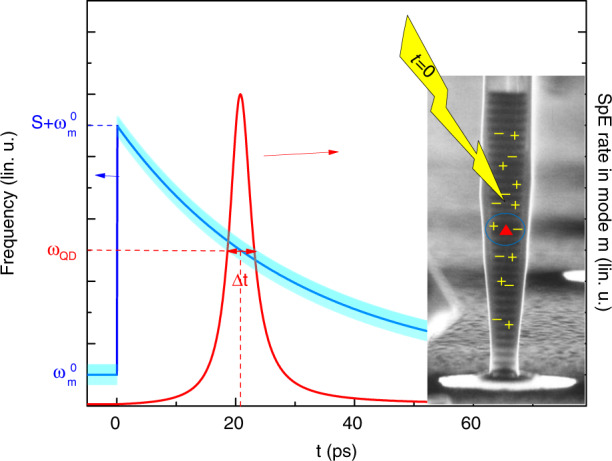
Sketch of principle of a Purcell switching experiment for a single QD in a pillar microcavity. (Right) The electron micrograph shows a typical QDM used in this work; here the pillar diameter is 1 µm. At *t* = 0, a laser pulse generates free charge carriers, which both excite the QD and induce a modification of the refractive index of GaAs, hence a frequency shift *S* of the cavity mode. (Left) Temporal dependence of the mode frequency ω_*m*_ (blue line, broadened by the mode linewidth Δω_*m*_). The QD emission frequency ω_QD_ belongs to the frequency interval [$\omega _m^0,\omega _m^0 + S$] that is swept by the mode during its relaxation. For the chosen parameters (35 ps relaxation time, *ħS* = 4 meV, $\hbar \omega _{{QD}} - \hbar \omega _m^0$ = 2 meV), resonant coupling between mode *m* and the QD occurs around 20 ps. Thanks to the Purcell effect, a few ps long SpE pulse is emitted into the cavity mode. The pulse intensity, shown by the red curve, is calculated using a rate equation model^25^.

An artificial atom in a switched optical microcavity is expected to experience the Purcell effect in a transient way, as sketched in Fig. 4. In spite of its simplicity, a rate equation analysis is very useful to figure out whether, and how strongly, signatures of Purcell enhancement are observable in such experiments^25^. We introduce here some important lessons from rate equation modeling that will be used in section “Preparation of ultrashort low-coherence pulses of SpE using switched QDM” to support the analysis of our experimental data.

We consider a single QD that is embedded in a pillar microcavity and located at the antinode of the fundamental cavity mode, hereafter named mode *m*. The mode frequency at rest is $\omega _m^0$, the mode linewidth Δ*ω*_*m*_ and the Purcell factor *F*_*p*_. We assume that the collection set-up captures entirely the radiation pattern of mode *m* in the far-field (collection efficiency *η*_*m*_ = 1) and a much weaker fraction *η*_*nr*_ of the QD emission into the continuum of non-resonant modes. For sake of simplicity, we consider that mode *m* is rapidly switched towards the frequency $\omega _m^0 + S$ at around *t* = 0 and that the QD is prepared into its excitonic state (for instance using π-pulse resonant pumping) at the same moment. The QD bandgap is at frequency ω_QD_ which belongs to the frequency interval [$\omega _m^0,\omega _m^0 + S$] that is swept by mode *m* during its relaxation towards its original frequency $\omega _m^0$. Finally, we assume that free carrier recombination and mode relaxation follow a common exponential law, defined by the switch-off time constant *τ*_off_.

With these assumptions, the exciton population, *P*_*x*_*(t)*, the time-dependent SpE enhancement factor, *F(t)*, and the collected PL intensity, *I*_*s*_*(t)*, satisfy this following set of equations:

1 $${d}P_x/{d}t = - P_x \cdot \left( {\frac{\gamma }{{\tau _{b}}} + \frac{{F(t)}}{{\tau _{b}}}} \right)$$

2 $$I_s(t) = \frac{{P_x}}{{\tau _{b}}} \cdot \left( {\gamma \eta _{{nr}} + F(t)\eta _m} \right)$$

3 $$F\left( t \right) = F_{p} \cdot \frac{{{{{\mathrm{{\Delta}}}}}\omega _m^2}}{{{{{\mathrm{{\Delta}}}}}\omega _m^2 + \left( {\omega _m\left( t \right) - \omega _{{QD}}} \right)^2}}$$

where $\tau _{b}$ is the exciton lifetime for a QD embedded in bulk GaAs and $\gamma /\tau _{b}$ the SpE of the exciton into the continuum of non-resonant modes.

For small-diameter micropillars in the 1–1.5 µm range, various *Q*’s ranging from 1000 up to 200,000 have been reported^1–3,37,38,66,67^, depending upon the numbers of GaAs/AlAs layer pairs forming the Bragg reflectors and, more importantly, upon the roughness of the pillar sidewalls, which is induced by the etching step^57^. We choose for this numerical study experimentally relevant parameters. We take as QD parameters $\hbar \omega _{{QD}} = 1.36\;{{{\mathrm{eV}}}}$ and $\tau _{b} = 1.3\;{{{\mathrm{ns}}}}.$ We fix the pillar diameter as 1.5 µm and its resonance frequency at rest as $\hbar \omega _m^0 = 1.358\;{{{\mathrm{eV}}}}$. We consider both moderate*-Q* (*Q* = 1000, *F*_*p*_ = 7) and high*-Q* (*Q* = 20,000, *F*_*p*_ = 136) micropillars and take γ = 0.9. As discussed in Gayral et al. ^68^, a key parameter in the context of the analysis of the PL intensity is the set-up collection efficiency, $\eta _{{nr}}$, for SpE that is emitted into the background non-resonant modes. $\eta _{{nr}}$ is usually very small for pillar microcavities, except for narrow pillar in the 1 µm diameter range, for which values around 0.1 have been reported. Larger values of $\eta _{{nr}}$ can be obtained for microdisk and photonic crystal cavities, depending on the collection geometry of the experimental set-up. To be more general, we chose therefore for this study $\eta _{{nr}}$ equal to 0.1, 0.2, or 0.5. Finally, the switching amplitude is chosen to be *S* = 6 meV and we assume for sake of simplicity an exponential relaxation of the mode, with time constant $\tau _{{off}}$ = 70 ps.

With these parameters, one expects the perfect resonance between the QD and the shifting cavity mode to occur around 80 ps. The most obvious signature of this transient coupling stems in the generation of a few-ps-long pulse of light by the QDM. The duration of this pulse ${{{\mathrm{{\Delta}}}}}t$ is simply given by the ratio of the mode linewidth and the shifting speed of the mode in the frequency domain. For a single exponential decay, one gets

4 $${{{\mathrm{{\Delta}}}}}t = \frac{{\tau _{{off}}}}{Q} \cdot \frac{{\omega _m^0}}{{\omega _{{\mathrm{QD}}} - \omega _m^0}}$$

Higher*-Q*’s lead to shorter interaction times, for a given shifting speed of the mode. Additionally, the detuning between the QD and mode frequency at rest $\omega _m^0$ appears as a simple tuning parameter for the pulse duration. The relaxation becomes slower as the mode frequency gets closer to $\omega _m^0$, which increases the interaction time for a QD close to $\omega _m^0$. These properties will be exploited in section “Preparation of ultrashort low-coherence pulses of SpE using switched QDM” to generate ultrashort SpE pulses.

**Fig. 5:**
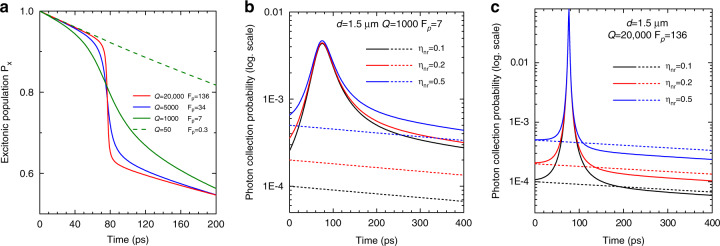
Rate equation modelling of on-off Purcell switching for a single QD in a QDM. **a** Time-dependence of the excitonic population in the QD for four different sets of (*Q*, *F*_*p*_) cavity mode figures of merit. **b**, **c** Solid lines show the evolution of the photon collection probability per pulse and per ps in log scale (this quantity is proportional to the PL signal in experiments) for a moderate*-Q* (1000 for **b**) and a high-*Q* (20,000 for **c**) pillar mode. We consider three different values of $\eta _{{nr}}$, ranging from 0.1 to 0.5. Dashed lines show the result for a reference emitter that would experience a negligible Purcell effect.

Another signature of the transient Purcell effect is the step-like behavior of the excitonic population, observed at around 80 ps in Fig. 5a. This step results from the accelerated decay of *P*_*x*_ induced by the QD-mode coupling. Its amplitude Δ*P*_*x*_, which quantifies also the probability for the QD to emit a photon in the mode during the transient coupling event, is approximately given by

5 $${{{\mathrm{{\Delta}}}}}P_x = \frac{{F_{p}}}{{\tau _{b}}} \cdot {\Delta}t = \frac{{F_{p}}}{Q} \cdot \frac{{\tau _{{off}}}}{{\tau _{b}}} \cdot \frac{{\omega _m^0}}{{\omega _{{QD}} - \omega _m^0}}$$

Interestingly, Eq. 5 predicts that, for a given pillar radius, Δ*P*_*x*_ does not depend upon *Q*. This is confirmed by the simulation results shown in Fig. 5a, obtained for various *Q*’s (Δ*P*_*x*_ is around 0.25, as predicted using Eq. 5). If one increases the cavity *Q*, one increases the Purcell factor but one decreases by the same ratio the interaction time between the mode *m* and the QD. In turn, the total SpE in the mode is roughly independent of *Q* (or *F*_*p*_). Measuring the amplitude of the population step can be used to prove the involvement of the Purcell effect in a switching experiment, but is not an adequate strategy if one aims at evaluating the maximum magnitude of the Purcell effect during the coupling event.

In principle, the cleanest way for probing experimentally the population decay is detecting selectively the emission into the background of non-resonant modes^25^. For in-plane emitting cavities, such as microdisks or cavities in 2D photonic crystals, one can relatively easily collect this emission in normal incidence. This is not the case for micropillars, whose emission spectra are usually overwhelmingly dominated by the signal related to cavity modes. In high-quality micropillars with smooth and straight sidewalls, most of the non-resonant emission is indeed guided in the GaAs/AlAs cylinder and escapes toward the substrate. As a result, it is generally not possible to detect population changes in a PL experiment in normal incidence on switched QD-micropillars (SQDM) as shown in Fig. 5b, c. For moderate*-Q* modes (*Q* = 1000), emission at long delays comprises emission of the QD into the tails of the LDOS of the relaxed mode. In the high-*Q* case (here 20,000), the observation of a clear drop of the PL signal after the coupling event would require values of $\eta _{{nr}}$ $\left( {\eta _{{nr}}/\eta _m \,>\, 0.1} \right)$ that are obtainable for in-plane emitting cavities but too large for high-quality micropillars.

For these reasons, it is safer to use the enhanced amplitude of the PL signal as a witness of the Purcell effect in experiments on SQDM. When compared to the decay of a far-detuned QD that would not be coupled to any mode, the PL intensity is typically enhanced by a factor

6 $$1 + \frac{F}{\gamma }\frac{{\eta _m}}{{\eta _{{nr}}}}$$

We recognize in this formula two combined benefits of the Purcell effect: SpE acceleration $\left( {F \,>\, \gamma } \right)$ and SpE funnelling into the cavity mode enabling better collection of emitted photons $\left( {\eta _m \,>\, \eta _{{nr}}} \right)$. A quantitative analysis of experimental data therefore requires prior knowledge of $\eta _{{nr}}$.

The on–off switching of the Purcell effect could be used, in principle, to trigger the emission of a single photon at a chosen time, from a QD that has been previously prepared in its single exciton state through resonant pumping. In the context of deterministic sources of indistinguishable single photons, this controlled delay would permit to filter-out the single-photons from scattered pump light by time gating. This approach looks advantageous compared to present protocols that rely on cross-polarization schemes for the pump and single-photon collection channels, and induce a strong penalty in terms of source efficiency^14,15^. Obviously, standard spectral filtering could be used to remove the (far-detuned) parasitic signals induced by the switching beam such as scattered light or GaAs luminescence. Alternatively, non-local switching could also be implemented to ensure the purity of the single-photon pulses as discussed in more detail in section “Some perspectives related to this work”.

As already mentioned, the efficiency of such a triggered source of single photons is given by Eq. 5. It could be boosted up to values close to unity by increasing the interaction time between the mode and the QD (while keeping *F*_*p*_ constant), either by increasing *τ*_off_ through improved surface passivation, or more easily by decreasing the detuning $\omega _{{QD}} - \omega _m^0$. Apart from micropillars, small volume cavities such as photonic crystal cavities look attractive in this context, as they enable to get a larger *F*_*p*_ for given values of *Q* and of the interaction time. Although very useful in practice, a rate equation analysis does not allow to go beyond the study of average exciton and photon populations. In the context of single-photon pulse shaping, refined modeling using quantum Langevin equation permits both to take into account decoherence effects and to estimate key figures of merits such as the fidelity with respect to a target pulse or the factor of indistinguishability of the single photons^53^.

**Fig. 6:**
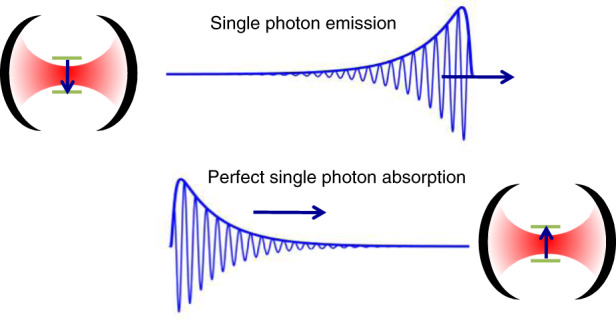
Sketch of ideal single-photon emission and absorption processes. (Top) An atom placed inside a static single-mode cavity emits a single photon with asymetric temporal envelope. (Bottom) Perfect single photon absorption by the atom in the cavity requires the single photon to possess a reversed temporal envelope.

Artificial atoms in a static photonic environment experience an exponential decay. Therefore, single-photon wave packets have a strongly asymmetric temporal envelope, with a step-like rise and an exponential tail whose decay is defined by the radiative lifetime. In the context of quantum photonics, such a shape is far from being optimal as shown by two emblematic examples. Firstly, it has been shown that Gaussian pulses are the best suited for linear optics quantum computing, as they enable to minimize the impact of arrival time jitter on the fidelity of two-photon gate operations^27^. Secondly, the asymmetric exponential envelope hinders perfect reabsorption by another emitter, even if it is strictly identical to the one that has emitted the single photon^28^. The success probability for the teleportation of a quantum state using single photon exchange between two atoms is at most 63%. Perfect reabsorption requires mimicking the time-reversal of a SpE event; in other words, the single photon should have an inversed exponential envelope as sketched in Fig. 6.

A widely used approach toward single-photon time-envelope shaping is post-emission amplitude modulation using electro-optical modulators^69–71^. In such experiments, pulse shaping is obtained at the price of some absorption, i.e., some reduction of the efficiency of the single-photon source. We have shown theoretically^53^ that both Gaussian and inverse-exponential pulses could be emitted with both a high efficiency and high fidelity with respect to the target pulse using a single QD in a high *F*_*p*_ microcavity. For this, free carrier switching of the cavity mode would be used to tune in real time the QD-mode detuning, hence the magnitude of the Purcell effect. The ultrafast response of cavity modes to all-optical free carrier injection is a key advantage, especially for the generation of inverse-exponential pulses, which is one of the most demanding applications in terms of Purcell effect tuning^53^.

**Fig. 7:**
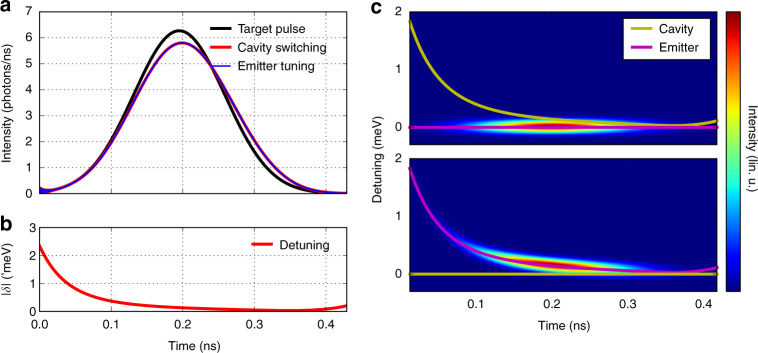
Modelling the generation of single photons with Gaussian temporal envelope. **a** Single photon temporal envelopes for the target Gaussian-pulse with 150 ps FWHM (black line) and pulses emitted by a realistic QD–cavity system using ultrafast emitter tuning (blue line) or cavity switching (red line). The QD is ideally located at the antinode of a photonic crystal cavity characterized by *Q* = 7000, *F*_*p*_ = 80 and *γ*_*nr*_ = 0.05. **b** Time-dependence of the detuning that is used to generate these single-photon pulses. **c** Time-resolved spectrum calculated using quantum Langevin equation for single-photon pulses obtained using cavity switching (top) or emitter tuning (bottom). A pronounced chirp is observable for the emitter-tuning approach.

One could naively think that changing the emitter frequency or the cavity mode frequency would lead, if both possible in practice, to strictly equivalent results. As shown in Fig. 7, this is only true for the temporal envelope of the pulse. In the weak coupling regime, the emission of the coupled atom–cavity system remains at all times close to the emitter frequency *ω*. As a result, emitter tuning leads to chirped single-photon pulses that are unsuitable to efficient reabsorption by another atom^30,53^. For this very reason, cavity switching is by far superior to emitter tuning for single-photon pulse shaping. Unlike emitter tuning, cavity switching has a negligible influence on the photon spectrum. It could allow, for realistic cavity parameters, to create Gaussian envelope single photons with a fidelity to the target photons of 99%, as well as single-photon with time-reversed envelopes, enabling reabsorption by an atom in a cavity with a probability well above 90%^53,72^.

## Preparation of ultrashort low-coherence pulses of SpE using switched QDM

As shown in the previous section, one can use cavity switching to induce a transient coupling between an artificial atom such as a QD and a cavity mode. Due to Purcell enhancement of the SpE rate into the cavity mode, the QD–cavity system emits a few-ps-long pulse of light.

One can extend this approach to cavities containing ensembles of artificial atoms. This is obvious for emitters such as rare-earth atoms^73^ or G-center defects in silicon^74^, whose ensembles exhibit a small inhomogeneous line broadening. By contrast, when emission wavelengths are dispersed, as for self-assembled QDs^75–77^, one has to rely on the spectral selection of a sub-ensemble of emitters, emitting all within a chosen narrow spectral window. The duration of the SpE pulse is then simply given by

7 $${\Delta}t = \sqrt {{\Delta}\omega _m^2 + w^2} /\frac{{{d}\omega _m}}{{{d}t}}\left( {t_{{res}}} \right)$$

in the general case, and

8 $${\Delta}t = ^{\tau_{{off}}}/\left( {\omega _{{QD}} - \omega _m^0} \right) \cdot \sqrt {{\Delta}\omega _m^2 + w^2}$$

when the relaxation of the mode is described by an exponential law, where *w* is the width of the frequency window, and *t*_*res*_ the time at which the mode frequency *ω*_*m*_ corresponds to the central frequency *ω*_*w*_ of the collection window.

**Fig. 8:**
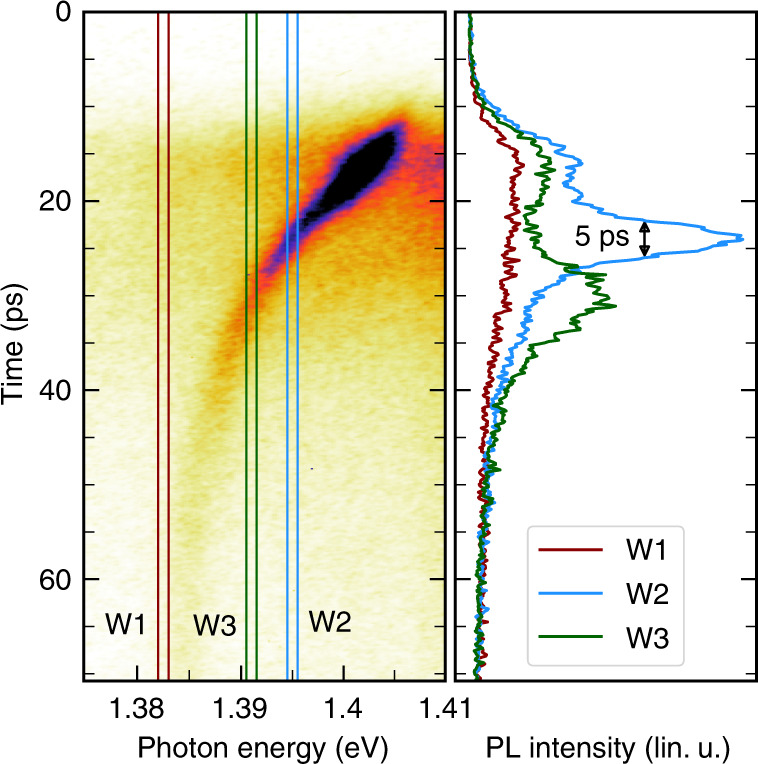
Emission of a few ps light pulse by frequency-selected QDs in a switched QDM. The streak camera image is obtained for a 1.6 µm diameter switched QDM (Q = 2000), and temporal profile of the PL signal that is collected for three 1 meV-wide frequency windows, positioned below (1.383 eV for W1), or above (1.395 eV for W2, 1.391 eV for W3), the frequency of the fundamental mode in the unswitched QDM $\hbar{\omega_m^\circ}=1.384\,{\rm{eV}}$. A single pump pulse at around *t* = 0 ps is used to excite and switch the QDM. Switching conditions are defined by *E*_*p*_ = 1.7 eV, *P* = 200 pJ/pulse; the switching amplitude is around 20 meV. The same linear scale is used for the three PL profiles. Please note that in this photon energy range, the emission is predominantly related to optical transitions between excited states of the QDs. The lower energy tail of the emission of the wetting layer may also contribute to the PL signal above 1.4 eV, under such strong pumping conditions (see SI).

We show in Fig. 8 the results of a proof-of-principle experiment aiming at showing how very short pulses can be generated following this procedure. We study here a 1.6 µm-diameter micropillar, for which a significant fraction of QD emission into non-resonant modes can be collected (*η*_*nr*_ is around 5%, see SI). On the basis of Eqs. 4 and 6, we also choose switching conditions that provide a large switching amplitude *S*, and therefore a large shifting speed for mode HE_11_ in the frequency domain. More precisely, we use a higher pump photon energy *E*_*p*_ (1.7 eV), so as to increase the density of electron–hole pairs in GaAs at saturation. A switching amplitude *S* as large as 20 meV is observed (which corresponds to a relative frequency shift Δ*ω*_*m*_/*ω*_*m*_ larger than 1%). Unlike experiments reported in section “Probing switching events with QDs as an internal light source”, a single pump pulse is used in this experiment to prevent emission from QDs prior to the switching pulse, and the emission behaviors of different collections of frequency-selected QDs, which do not enter (for window W1), or do enter (for windows W2 and W3), into resonance with the mode during its relaxation towards its frequency at rest, are compared. We obtain the time-resolved PL profile for QDs in each window directly from the streak camera image, through an integration at all times of the signal collected within that frequency window. As shown in Fig. 8, we observe, for all windows and for time delays between 0 and 15 ps, the rise of the QD PL that is emitted into the continuum of non-resonant modes of the QDM. For W1, the PL signal decays in a monotonic way at longer time delays. Such a behavior is thus qualitatively similar to the one of QDs in an unswitched micropillar (see SI). By contrast, the temporal PL profile exhibits a sharp additional peak (5 ps FWHM), at around a 25 ps delay for window W2, due to the transient coupling of the QDs to the mode. When resonance is reached, the collected signal is increased typically by a factor of 3 with respect to the baseline of the peak, which is related to emission into non-resonant modes. This specific feature reflects the on–off switching, on the few-picoseconds time scale, of the coupling between QDs emitting at energies within W2 and the HE_11_ cavity mode.

A similar behavior is observed for QDs emitting in window W3. However, the additional peak is observed at a longer delay after the pump pulse (30 ps), and its duration is slightly longer (around 9 ps FWHM) than for W2. Since W3 is closer to the unswitched mode frequency $\omega _m^0$ than W2, the mode enters indeed into resonance with W3 at a later stage of its relaxation, and crosses window W3 at a reduced shifting speed.

At this stage, we have not yet probed the nature of these short pulses of light. Since the QDM contains many QDs (~ 6000 for a 2 µm diameter), besides SpE, stimulated emission could contribute to the generation of the light pulses when the mode enters into resonance with the QDs in windows W2 or W3.

**Fig. 9:**
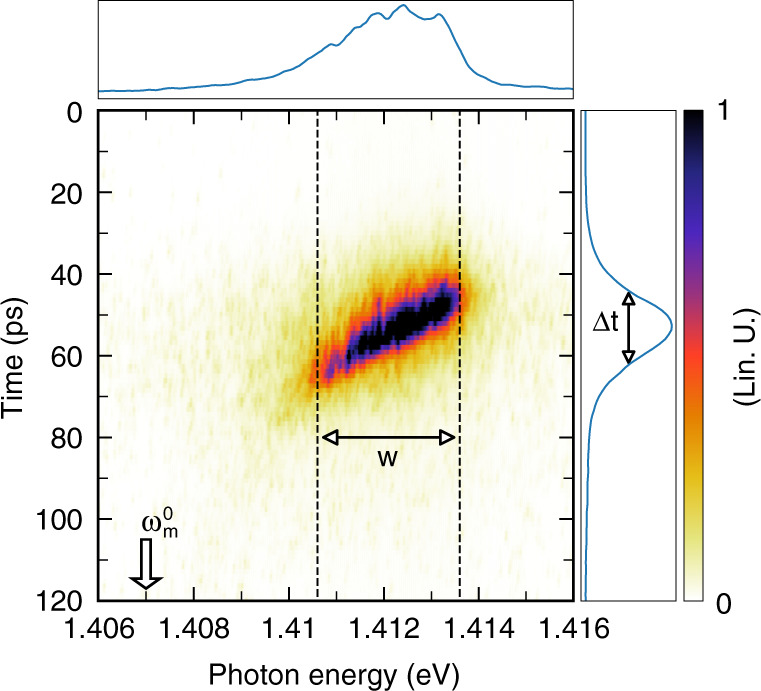
Temporal and spectral analysis of the light pulses emitted by QDs in a switched QDM. Streak camera image obtained for light pulses emitted by a sub-ensemble of QDs in a switched QDM that have been frequency-selected using a pass-band filter (2μm diameter, $\hbar{\omega_m^\circ}$ = 1.407 eV, $\hbar{\omega_w}$ = 1.412 eV, w = 3 meV), during its transient coupling with the fundamental mode of the cavity. The pump beam is matched to the top facet of the QDM, *E*_*p*_ = 1.6 eV, *P* = 50 pJ/pulse and *S* = 8 meV. The PL spectrum (top panel) and the temporal PL profile (right panel) are shown in linear scale, and have been, respectively, obtained from the bare image by summing the PL signal over all times and all photon energies.

We have therefore studied the temporal coherence of the pulses produced by the switched QDMs, to test whether SpE or stimulated emission is at play for their production. In view of this complementary experiment, we use a different scheme for QD frequency-selection. We select here the emission of QDs within a chosen frequency window using bandpass filters. We expect, according to the results of our proof-of-principle experiment, that a short light pulse escapes from the filter, each time the QDM is pumped. We confirm the successful preparation of the pulse, and study its properties using the streak camera (see Fig. 9). In this experiment, the window width *w* is around 3 meV, so that the pulse duration is around 16 ps. *w* is also larger than the mode linewidth (0.5 meV); in turn, light pulses display some chirp, because, within the frequency window, QDs at higher energy enter into resonance with the cavity mode earlier than QDs at a lower energy.

If only SpE is at play, all QDs within the spectrally selected sub-ensemble behave as independent emitters, each emitting at different frequencies. Looking at Fig. 9, we see that the pulses are well above the Fourier limit (*w* . Δ*t* ≈ 100 » 1). Assuming they stem from the sum of uncorrelated SpE from numerous QDs spread all over the collection window, one expects a coherence time $\tau _{{coh}}^0$ shorter than 1/*w* ≈ 0.12 ps and a coherence length of about 35 µm. (One has to note that these values give only upper bounds for these quantities, since the coherence time is additionally reduced by the jitter on QD pumping, as well as dephasing processes that impact single QD emission^78–80^ under non-resonant pumping conditions).

We have probed in a qualitative way the coherence of the pulses emitted by switched QDMs by studying their transmission through a 500 µm Teflon film, used as scattering medium. At around 1.4 eV, the photon mean free path is around 50 µm^81^, i.e., much smaller than the thickness of our film, so that light experiences many scattering events before escaping at the exit side. For a large enough coherence length of the impinging beam (i.e., larger than the optical length difference for paths resulting from different scattering events), one expects the formation of a 2D speckle pattern at the output facet of the scattering medium^82^. For this experiment, we use an imaging system based on a Si charge-coupled device (CCD) camera to probe the appearance of speckles^54^ (see also SI). As a reference source, we use 3 ps long laser pulses at *ħω* = 1.4 eV. Since *L*_coh_ is much larger than the film thickness for such laser pulses, a clear speckle pattern is observed^54^. This pattern is identical for two independent acquisitions, and does not depend upon pulse energies, as expected^82^.

**Fig. 10:**
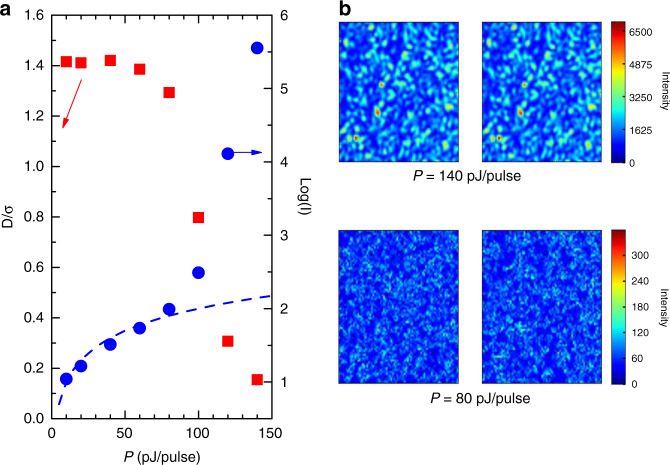
Qualitative testing of the spectral coherence of light pulses generated by a switched QDM. **a** Evolution of the indicator of speckle formation *D/σ* and of the intensity *I* of the pulses delivered by the switched QDM micropillar (blue dots) as a function of the pump intensity. For *P* below ≈70 pJ/pulse, *I* follows the linear trend expected for pure SpE, shown as a dotted blue line. For larger *P*’s, a superlinear increase of *I* reveals the onset of amplification by stimulated emission from the QD ensemble. **b** CCD camera images of the output facet of a Teflon film, upon irradiation by pulses emitted by the switched micropillar for two different *P*’s. For each energy, we show two images obtained from independent acquisitions. Speckle formation is clearly observed in the strong pumping regime.

We perform this experiment with the QDM light pulses, whose analysis is shown in Fig. 9. For this QDM, the switching amplitude *S* is larger than 6 meV for pump powers larger than 10 pJ/pulse. Therefore, light pulses can be prepared using the same frequency window and different pump intensities *P* (here, ranging between 10 and 140 pJ/pulse). We show in Fig. 10 pairs of images obtained from independent runs for two different *P*’s. In order to help us detect speckle formation, we use two numerical indicators. Denoting *C*_*nm*_ the number of counts for the CCD pixel (*n*,*m*) and $\bar C$ the average number of counts, the standard deviation σ, which reflects intra-image fluctuations of the normalized pixel intensity, is given by

$$\sigma ^2 = \mathop {\sum }\limits_{nm} \left( {C_{nm} - \bar C} \right)^2/\bar C^2$$

We also define an inter-image mathematical distance *D*, to estimate how different two images are from one another:

$$D^2 = \mathop {\sum}\limits_{nm} {\left( {\frac{{C_{nm}}}{{\bar C}} - \frac{{C_{nm}^\prime }}{{\overline {C{^{\prime}} } }}} \right)^2}$$

where *C*ʹ_*nm*_ is the number of counts for pixel (*n*,*m*), and $\overline {C{^{\prime}} }$ the average value of *C*ʹ_*nm*_ for the second image. Trivially, *D* = 0 for two identical images; more generally, for highly contrasted and strongly correlated speckled images (large σ, small *D*), one expects *D/σ* to become vanishingly small. By contrast, one can easily show that for unstructured images for which deviations of pixel intensities with respect to the average value are only due to random noise (no inter-pixel correlations), then $D/\sigma = \sqrt 2$.

As shown in Fig. 10, this criterion is very nicely satisfied for the pair of images obtained for *P* = 60 pJ/pulse, as well as for smaller *P*’s. Additionally, σ is close to the expected value for inter-image intensity fluctuations related to the shot noise. We can thus conclude that the light pulses that are prepared for *P*’s in the 10–60 pJ/pulse range are SpE pulses. This is additionally confirmed by the study of the intensity *I* of the light pulse, which shows a linear dependence as a function of the pump pulse energy *P* within this power range.

By contrast, we observe for this QDM a strong non-linear dependence of the intensity of the emitted pulses as a function of *P* for values above 70 pJ/pulse. We attribute this behavior to amplification by stimulated emission. At high pump powers, one expects a saturation of the QDs’ ground states and a filling of their excited states as observed for reference QD layers grown under similar conditions (see SI). As the frequency window W is located at around 1.41 eV, in the high-energy tail of the distribution of our QDs, this filling increases the number of QDs that emit within W and allows reaching the lasing threshold.

The transmission experiment through Teflon reveals a radically different behavior for pulses prepared in this strong pumping regime as shown in Fig. 10 for *P* = 140 pJ/pulse. CCD images resulting from different acquisitions look very similar and “hot spots” are clearly visible. Speckle formation is confirmed by considering the two criteria introduced above. On one hand, the pixel to pixel intensity fluctuation *σ* is, for both images, 250 times larger than the shot noise level. On the other hand, *D*/σ = 0.12 ≪$\sqrt 2$, which highlights the high degree of resemblance between these two highly contrasted images. These results show that the coherence length of SQDM pulses increases drastically when going from low to high power pumping regimes, due to the onset of stimulated emission. The thresholds for amplification by stimulated emission and for speckle formation are precisely in line: coherence-free pulses are only generated by SQDMs in the SpE regime, a crucial point for potential applications.

In this experiment, we have shown that 16 ps-long pulses of SpE can be produced by spectrally filtering the emission of a switched QDM. It is interesting to note at this point that short SpE pulses can of course be emitted by QD ensembles in static QDMs in the Purcell regime. However, their duration cannot be shorter than around 25 ps, even for giant Purcell factors (*F*_*p*_ > 100), because of the time jitter induced by carrier capture and relaxation times under non-resonant pumping conditions^83,84^. Cavity switching appears as an utmost new approach to generate short light pulses in a controlled way. As an additional illustration, we show in the SI that a wide range of pulse durations (from about 500 ps down to 5 ps), can be obtained using switched QDMs in the SpE regime, by simply varying the window detuning *ω*_*w*_ − *ω°*_*m*_.

## Some perspectives related to this work

The results that have been presented in this paper open interesting perspectives in two main domains: ultrafast imaging and single-photon pulse shaping for quantum photonics.

The interest of low-coherence light pulses for imaging is well recognized, as they enable, e.g., detection of objects hidden by a turbid environment^85–87^ or obtaining clear images of ultrafast processes in fluids^88^. Laser pulses are indeed poorly adapted to such applications, as their coherence induces diffraction fringes and speckle patterns in recorded images. State-of-the-art low-coherence sources exploit few ns long fluorescence pulses emitted by a laser dye^88^. SQDMs could in principle be used to extend ultrafast imaging to the sub-ns range, which is of interest for various domains (fluidics, shock wave propagation among others). At first sight, though, SQDMs suffer from their small power. Their mere operation principle limits the average photon number per pulse to Δ*τ/τ*_cav_, so as to avoid stimulated emission, e.g., 10 photons/pulse for *Q* = 1500 and Δ*t* = 10 ps. By using longer pulses (Δ*t* ~ 200 ps) and operating a large array (100 × 100) of very similar SQDMs in parallel, one would reach 2 × 10^6^ photons/pulse. Though challenging, this strategy is likely to open a route towards the imaging in a stroboscopic mode of highly reproducible processes such as inkjet droplet formation^88^ on a sub-ns time scale. Thanks to its high repetition rate (76 MHz here, with potential increase up to 5 GHz), a single SQDM could deliver up to 10^11^ photons/second. Therefore, the detection of objects hidden in turbid media such as fog or murky water can be considered a realistic target in the short term.

As mentioned in section “Controlling SpE dynamics using cavity switching: insights from theory”, cavity switching could be of interest in the quest for efficient triggered sources of indistinguishable single photons. Furthermore, the shaping of the temporal envelope of indistinguishable single-photons emitted by single QDs looks within reach in the mid-term using state-of-the-art optical microcavities. Nevertheless, major additional developments will be necessary to reach these goals. In order to avoid repumping of the QD by carriers that are used for cavity switching (which would spoil the purity of the single-photon source), as well as dephasing induced by the Coulomb interaction of the QD exciton with free carriers (which would plague single-photon indistinguishability), some kind of non-local switching has to be performed as shown by several groups on photonic crystal cavities^44–47^. “Photonic molecules” made of coupled micropillars^3^ or coupled cavities in a photonic crystal membrane^46,47^ (or other extended cavities such as high-*Q* ring-resonators) are well suited for such experiments, where two synchronized lasers would be used to pump resonantly the QD on one side, and switch cavity modes (without pumping the region containing the QD) on the other side. A local engineering of the non-radiative recombination rate (using for instance ion implantation) could be developed to warrant a fast switch-off dynamics. The method introduced in section “Probing switching events with QDs as an internal light source” to characterize switching events might prove particularly helpful for the development of these sophisticated photonic microstructures.

## Supplementary information

Supplementary information for tailoring the properties of quantum dot-micropillars by ultrafast optical injection of free charge carriers
