# Supplementary material for: Tailoring the properties of quantum dot-micropillars by ultrafast optical injection of free charge carriers
Source: Light Sci Appl. 2021 Oct 19;10:215. doi: 10.1038/s41377-021-00654-y (PMC8526595; doi:10.1038/s41377-021-00654-y)
Supplement: Supplementary file 1 — Supplementary information for tailoring the properties of quantum dot-micropillars by ultrafast optical injection of free charge carriers [file 41377_2021_654_MOESM1_ESM.pdf]

Supplementary information for

Tailoring the properties of quantum dot-micropillars by ultrafast optical injection of free charge carriers

Emanuel Peinke,[1] Tobias Sattler,[1] Guilherme M. Torelly,[2] Patricia L. Souza,[2] Sylvain Perret,[1] Joël Bleuse,[1] Julien Claudon,[1] Willem L. Vos[3], and Jean-Michel Gérard[1*]

[1]Univ. Grenoble Alpes, CEA, IRIG-PHELIQS,"Nanophysique et semiconducteurs" group, F-38000 Grenoble, France

[2] LabSem-CETUC, Pontifícia Universidade Católica do Rio de Janeiro, 22451-900, Brazil

[3]Complex Photonic Systems (COPS), MESA+ Institute for Nanotechnology, University of Twente, P.O. Box 217, 7500 AE Enschede, the Netherlands

*jean-michel.gerard@cea.fr

Sample design, fabrication and characterization

Micropillars containing quantum dots (QDs) have been processed from a planar microcavity M, grown by molecular beam epitaxy. M is formed by a GaAs one-wavelength thick cavity layer, surrounded by a 15 (on top) and a 25 (at the bottom) period distributed Bragg reflectors (DBRs) made of quarter-wavelengths GaAs and AlAs sublayers. This planar microcavity is designed for an operation wavelength around $\lambda = 0.91$ µm at a temperature of 4 K, assuming refractive indices equal to 3.5 for GaAs and 2.95 for AlAs. Due to some non-uniformity of the growth rate across the wafer, the thickness of the cavity layer varies gradually across the sample and, in turn, so does the resonance frequency of the planar cavity under normal incidence excitation.

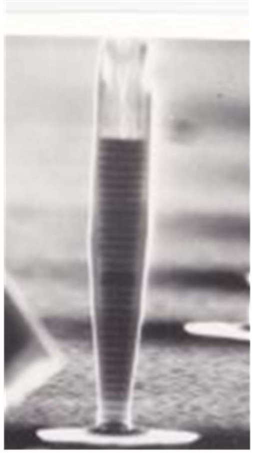

Fig. S1 Typical scanning electron micrograph showing the structure of QD-micropillars used for this study. The diameter of the top facet of this pillar is 1 µm. Image adapted from [SI1].

Five layers of InAs QDs (areal density ~4. 10^{10} cm^{-2} per layer) have been inserted within the cavity layer, close to the antinodes of the resonant

mode in the planar cavity. More precisely, two layers are located at 10 nm from each interface with the DBRs, and the other three are placed at the center of the cavity layer, 10 nm above its center, and 10 nm below it.

In order to form the InAs QD layers, a 0.6 nm equivalent thickness of InAs is deposited within 1 s at 520 °C, and the GaAs overlayer is immediately grown on top. With this procedure, small QDs are formed, not significantly disturbing the subsequent growth of the top DBR, and emitting at a relatively high energy [SI2]. The PL spectra obtained for a reference QD sample for different pump powers are shown in Fig. S2. For a pump power *P* sufficiently low to avoid a saturation of the fundamental optical transition of the QDs (here *P* = 10 µW), the emission of such a QD ensemble is spectrally broad due to QD size fluctuations (45 meV FWHM) and centered around 1.36 eV. For stronger pump powers, optical transitions between excited electron and hole states of the QDs contribute to the PL spectrum, broadening the high-energy side. The QDs emission band extends up to the bandgap of the wetting layer, that is observed at around 1.43 eV for such growth conditions [SI2]. Please note that we choose on purpose for our experiments such small QDs (emitting within the upper part of the spectral range that can be covered by InAs/GaAs QDs), because the sensitivity of the detection system increases significantly as a function of the photon energy in the near infrared (see below).

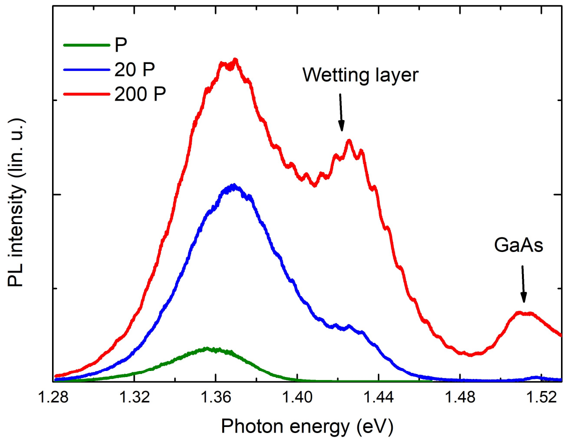

Fig. S2 PL spectrum obtained at 4 K for three different pumping powers, for a single layer of InAs QDs in GaAs, grown under the same conditions as the QDs layers embedded in the QDM used for switching experiments. The pump photon energy is 1.7 eV. (The small ripples on the high power spectrum are due to an interference effect related to the geometry of the sample).

The micropillar cavities have been processed according to the following procedure [SI3]. A 2 µm thick ''hard-mask'' layer consisting of baked optical resist is first deposited on the sample. Electron-beam lithography using polymethylmethacrylate and the lift-off technique are used to define a 100 nm thick Al mask, which is transferred to the hard-mask layer by reactive ion etching (RIE) using a SF_6 plasma. RIE using $SiCl_4$ is then performed to etch the epitaxial structure. As shown by the scanning electron micrograph shown in Fig. S1 for a 1 µm diameter micropillar, straight and smooth sidewalls are obtained. Micropillars with diameters ranging between 1 µm and 6 µm have been fabricated.

Micropillars have been first characterized by cw microPL, using the emission of embedded QDs as an internal broadband light source, as initially described in [SI4]. As shown in Fig. S3, one can extract from such an experiment the resonant frequencies as well as the quality factors, of a large number of resonant modes. The quality factor *Q* of their fundamental mode increases from 1000 to about 5000 as a function of the diameter, as previously reported for similar structures [SI3].

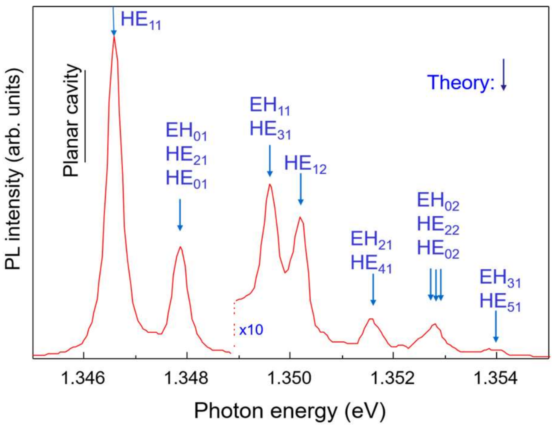

Fig. S3 PL spectrum obtained at 4K for a 5.7 µm diameter QDM under cw pumping. The pump photon energy (1.48 eV) is below the bandgap of GaAs to avoid mode shifts due to free carrier effects.

In the switching experiments reported in the main manuscript, a first pulse is used to light up the QD emission, in order to probe the modes properties just before and during the switch-on event. For several pillars, we have compared the PL spectrum (extracted from the streak camera image) just before the strong switching pulse to cw PL spectra. We do not observe any sizeable shift of the modes with respect to cw microPL data, which confirms that switching effects due to the first pulse are negligible.

Modelling of the mode frequencies

We identify the emission lines in the PL spectra of unswitched QDM, such as the one shown in Fig. S3, from a standard modelling [SI4]. Full confinement of light in a micropillar cavity results from the combination of waveguiding by the high index cylinder of GaAs/AlAs, and back and forth reflection by the Bragg reflectors. We start with a standard calculation of the effective indices of the guided modes of a 5.3 µm diameter cylinder in air [SI5]. Each guided mode *m* of the cylinder gives rise to a single resonant mode in the micropillar, whose resonance frequency ω_m is given by :

$$\omega_m / \omega_{2D} = n / n_m^{eff} \qquad \text{(S1)}$$

where *n* is the refractive index of undoped GaAs, ω_{2D} the normal-incidence resonance frequency of the planar cavity from which the micropillar has been fabricated and n_m^{eff} the effective index of the guided mode *m*. In Fig. S3, we use as labels for the pillar modes the usual names HE_{ij} or EH_{ij} of their ”parent” guided modes in the GaAs cylinder. Since the effective index of higher-order guided modes is smaller than the one of the fundamental guided mode, all higher-order resonant modes in the QDM are blue-

shifted with respect to HE_{11}. In spite of its simplicity, this model gives an excellent description of the frequencies of resonant modes in pillar microcavities (as shown e.g. in Fig. S3) with diameters in the few µm range.

We have recently extended this approach to the modelling of time-dependent mode frequencies in switched QDM [SI6]. We calculate first the transverse distribution of the free carriers in the cavity, taking into account the geometrical parameters of the Gaussian pump beam, the lateral diffusion of the carriers and their recombination at etched sidewalls. Since the cavity is relatively thin, we neglect the dependence of the carrier density as a function of the vertical position *Z*. We obtain for all times a map of the refractive index inside the perturbed cavity, assuming a linear variation of the refractive index change as a function of carrier density. We calculate the guided modes of the perturbed waveguide as well as their effective index, using a finite-element numerical approach (FEMSIM software, from Synopsys Corp.). We deduce finally the time-dependent frequencies of the resonant modes in the switched QDM using Eqn. S1. As shown by Fig. 2 and Fig. 3 in the main manuscript, a satisfying agreement can be obtained for all modes and all times, using a single adjustable parameter, which is the maximum amplitude of the refractive index change, induced just after the switching pulse at the center of the Gaussian pump beam.

Experimental set-up

The QD-micropillar sample is placed at a temperature around 4 K inside a helium-flow cryostat. A single micropillar at a time is excited by a Ti:sapphire laser delivering 200 fs long pulses at a 76 MHz repetition rate. We use an epi-microfluorescence experimental configuration, for which the same microscope objective (Zeiss 441030-9901, NA = 0.25) is used to focus the laser beam and to collect the QD-micropillar emission, which is shaped into a collimated beam.

In order to probe the mode frequencies before and during the switch-on, a two pump-pulses approach is followed [SI6]. A fibered system is used to split the pulses delivered by the Ti:sapphire laser into a weak pulse, which lights up the QDs but does not induce significant switching, and a stronger one, which is delayed by typically 100 ps with respect to the first one and switches the cavity.

For the characterization of the light emitted by switched QD-micropillars (SQMs), a system composed of a Jobin-Yvon Triax 320 monochromator and a Hamamatsu C10910 streak camera (S25 photocathode) is used. This system provides a temporal resolution around 2 ps, combined to a 0.3 meV resolution in the photon energy/frequency domain, close to the limit imposed by the time-energy

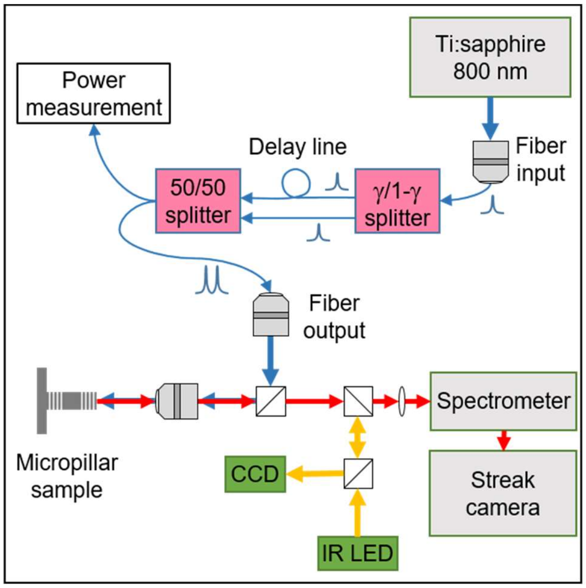

Fig. S4 Schematic view of the time-resolved microphotoluminescence set-up used for this work. The micropillar cavity is placed in a He-flow cryostat working at 4 K. Blue curved lines represent optical fibers. Blue arrows mark the excitation optical path; red arrows are for the detection path. An infrared light emitting diode (IRLED) and a CCD camera are used to visualize the sample while positioning the pump spot with respect to a micropillar top facet. The related optical paths are in yellow.

uncertainty principle. The sensitivity of the S25 photocathode increases rapidly as a function of photon energy in the 1.25 - 1.5 eV range. This is why the experiments are performed with SQM emitting in the 1.35 - 1.42 eV range, which corresponds to the high-energy part of the emission range covered by the InAs/GaAs QD system.

Over the latter photon energy range, the sensitivity of the photocathode increases by a factor of 5, according to the specifications of the manufacturer. The streak camera images were not corrected to take into account this effect, since it does not impact the conclusions about the temporal evolution of mode frequencies (Figures 1, 2 and 3 of the main manuscript), or the duration of pulses emitted by frequency-selected QDs in switched QDM (Figures 8 and 9 of the main manuscript).

Preparation and characterization of SQM pulses

For the test of the temporal coherence of SQM pulses, the emission of QDs is selected within a frequency window around 1.4 eV using band-pass filters (Semrock LP02-830RU-25). The collimated beam impinges on a 0.5 mm thick film of poly-tetrafluoroethylene (PTFE) (Goodfellow, ref. FP301400) under normal incidence and illuminates an area of about 1 mm^2. We image the opposite facet of the film on a CCD camera (model Coolsnap-ES from Roper Scientific) using a microscope objective. The images shown in Fig. 10 of the main manuscript correspond to a 50 µm x 50 µm area of the output facet, centered on the optical axis.

Tailoring the duration of SpE pulses emitted by a switched QDM

In this section, we show that the duration of SpE pulses emitted by frequency-selected QDs in a switched QDM can be adjusted over a wide time range, by playing only with the central frequency of

the selection window. We show in Fig. S5 some characterization results which have been obtained on the 3 µm diameter micropillar, that has been used for this experiment. When a pump photon energy below GaAs bandgap is used, one excites the QDs without switching the cavity. We observe the fundamental mode of the micropillar at a frequency ω_m^0 around 1.397 eV, with a *Q* around 3000. We see also that the QDs that are coupled to the mode experience a monotonous relaxation, as expected for emitters coupled to a static photonic environment.

Fig. S5 also shows a streak camera image obtained for a pump photon energy of E_p = 1.55 eV and a pump power around 30 pJ per pulse. In this case, we observe the switching of the fundamental cavity mode. The switching amplitude *S* is around 5 meV. The relaxation of the mode is accurately described by a bi-exponential law, with characteristic time constants 40 ps and 200 ps.

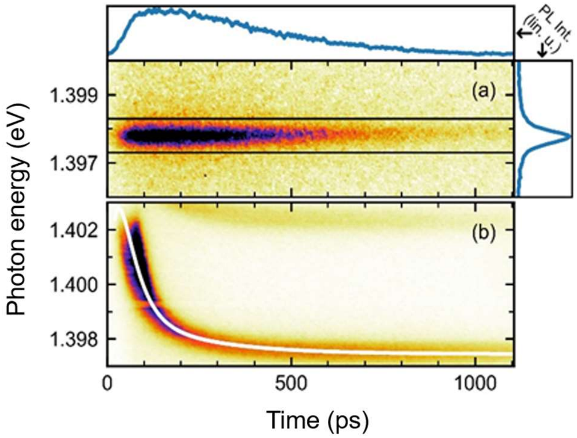

Fig. S5 a Streak camera image, PL spectrum (right curve) and PL decay profile (top curve) obtained for a 3 µm diameter QDM for E_p = 1.47 eV. **b** Streak camera image obtained for E_p = 1.55 eV and *P* = 30 pJ per pulse. The white line corresponds to a biexponential fit of the mode relaxation. The pump pulse is centered at t = 0 ps.

By exploiting the spectral resolution of our setup, we test the capability of this QDM to generate short SpE pulses when using frequency-selection of the QDs. We choose on purpose for this experiment a relatively low pump power (30 pJ per pulse), so as to avoid any amplification by stimulated emission. We select on the streak camera image the PL signal related to a sub-ensemble of QDs, emitting within a 0.3 meV wide frequency window. As shown in Fig. S6 for two different windows, the behavior of QDs in a switched QDM is drastically different from the monotonous decay observed for QDs in unswitched QDM. Temporal PL profiles exhibit an emission burst, related to the resonance of the QDs with relaxing mode HE_{11}. As explained in the main text, the duration Δt of this emission burst corresponds to that of the transient coupling between the QDs and the shifting mode, and is simply given by :

$$\Delta t = \sqrt{\Delta\omega_m{}^2 + w^2} \Big/ \frac{d\omega_m}{dt}(t_{res}) \qquad \text{(S2)}$$

As shown by this equation, Δt can be tuned by playing with the detuning of the frequency window ω_W with respect to ω_m^0, since the shifting speed of the mode $d\omega_m/dt$ increases with this detuning. As an example, the detuning ω_m^0 - ω_W is equal to 1 (5) meV for W1 (W2), leading to pulse durations (defined as the pulse width at half-maximum) around to 200 ps for W1 and as short as 6 ps for W2.

We plot in Fig. S6b the pulse durations deduced from this analysis. They cover approximately a two orders of magnitude wide time range, by only playing with the central window frequency ω_w. A modeling of burst durations based on the bi-exponential fit of ω_m(t) and Eqn. S2 accounts very well for these experimental results.

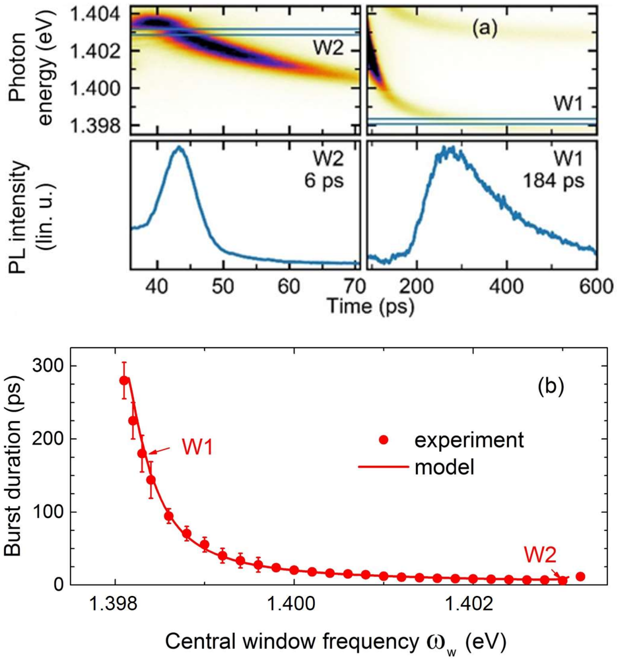

Fig. S6 a The top panels show streak camera images obtained for the fundamental mode of the same QDM as in Fig. S4 (E_p = 1.55 eV, P = 30 pJ per pulse). Two different time ranges are used for sake of clarity. Collection windows W1 and W2 are marked by horizontal solid lines. The bottom panels show the temporal dependence of the QDM emission within the frequency windows W1 and W2. **b** Measurements (dots) and theoretical estimate (line) of the duration of SpE pulses emitted in mode HE_{11}, as a function of the central window frequency ω_w (w = 0.3 meV).

Toward a high-resolution observation of mode crossings induced by a localized on axis perturbation.

In Fig. 2 c of the main manuscript, we show on the same figure the streak camera image and the result of a modeling of the temporal-evolution of the mode frequencies. We had the feeling that some readers could be interested by having access to the raw experimental image; it is shown in Fig. S7.

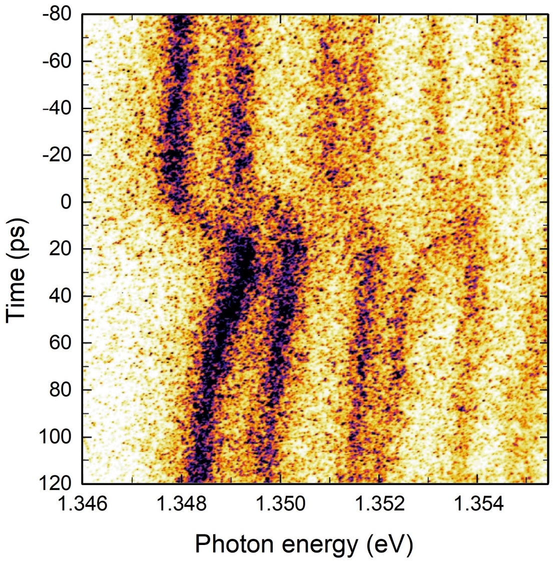

Fig. S7 Streak camera image obtained on a 6 µm diameter switched QDM excited by a focused centered pump beam. Here, E_p = 1.65 eV and P = 20 pJ per pulse. The Gaussian pump beam has a 2 µm diameter waist. The overlap of *Lines 4* and *5* is clearly observed around 10ps after the switching pulse.

References

[SI1] Gérard, J. M. et al. Enhanced spontaneous emission by quantum boxes in a monolithic optical microcavity. *Physical Review Letters* **81**, 1110-1113 (1998).

[SI2] Gérard, J.M. et al. Optical investigation of the self-organized growth of InAs/GaAs quantum boxes, *Journal of Crystal Growth* **150**, 351 (1995)

[SI3] Gérard, J. M. Solid-state cavity-quantum electrodynamics with self-assembled quantum dots. In Single Quantum Dots: Fundamentals, Applications, and New Concepts (ed Michler, P.) (Berlin, Heidelberg: Springer), *Topics in Applied Physics* **90**, 269 (2003)

[SI4] Gérard, J.M. et al. Quantum boxes as active probes for photonic microstructures: The pillar microcavity case, *Applied Physics Letters* **69**, 449 (1996)

[SI5] Yariv, A. Optical electronics (Saunders College, San Francisco, 1991)

[SI6] Sattler, T. Probing cavity switching events with an internal quantum dot light source, *APL Photonics* **5**, 126104 (2020)
